# Live-Cell Detection of Relative Intracellular Tension Dynamics Using Non-FRET α-Actinin and α-Catenin Tension Indicators

**DOI:** 10.21769/BioProtoc.5826

**Published:** 2026-10-05

**Authors:** Maretoshi Hirai, Keita Fujiwara

**Affiliations:** 1 Department of Pharmacology, Kansai Medical University, Hirakata, Osaka, Japan; 2 Department of Medicine II, Kansai Medical University, Hirakata, Osaka, Japan

**Keywords:** Molecular tension sensor, Mechanobiology, α-actinin, α-catenin, Live-cell imaging, Super-resolution microscopy, Blebbistatin, Cardiomyocyte, Ratiometric fluorescence imaging, Relative molecular loading, Chromatic registration

## Abstract

Molecular tension sensors enable the visualization of forces acting on specific intracellular proteins in living cells. Most established genetically encoded sensors rely on Förster resonance energy transfer (FRET), requiring donor–acceptor imaging and correction for spectral bleed-through, which can complicate their application in heterogeneous tissues. We developed non-FRET α-actinin and α-catenin tension indicators containing a force-responsive tension sensor (TS) module composed of an optimized circularly permuted enhanced green fluorescent protein (cpEGFP) scaffold and an elastic (GPGGA)_8_ linker. The TS module is incorporated into α-actinin or α-catenin, while a C-terminal mCherry serves as a force-insensitive reference for indicator abundance. This protocol describes indicator expression in cultured cells, validation using the myosin II inhibitor blebbistatin, two-color time-lapse imaging and live-cell super-resolution imaging using SRRF-Stream reconstruction based on super-resolution radial fluctuations (SRRF), and calculation of the green/red fluorescence ratio and normalized relaxation ratio. The protocol also specifies essential quality-control procedures, including imaging under nonsaturating conditions with fixed channel-specific acquisition settings across comparisons, registration of the green and red channels, and the use of appropriate vehicle and negative controls. For SRRF-Stream analysis, the green and red channels are reconstructed separately using identical reconstruction settings before ratio calculation. Representative reconstructed images should be compared with the corresponding conventional images to check for reconstruction artifacts. Optional procedures describe imaging of isolated cardiomyocytes and freshly isolated organs from tension-indicator mice. Because molecular loading is inferred from changes in TS fluorescence normalized to mCherry rather than from energy transfer between two fluorophores, the method avoids FRET-specific bleed-through correction; nevertheless, controlled acquisition and channel registration remain necessary. The indicators report relative changes in molecular loading and are particularly useful for resolving protein-specific and subcellular heterogeneity in tension dynamics.

Key features

• Genetically encoded, FRET-free tension sensor module reports relative changes in forces acting on α-actinin or α-catenin.

• C-terminal mCherry provides an internal reference for indicator abundance and motion-related intensity changes.

• Quantitative ratios are calculated from conventional 16-bit images (0–65,535) using ROIs with signals above local background in both channels and no saturated pixels.

• Validated in MDCKII and NIH3T3 cells and cardiomyocytes from genetically targeted mice, the protocol supports routine confocal time-lapse imaging and optional SRRF-Stream reconstruction.

## Graphical overview


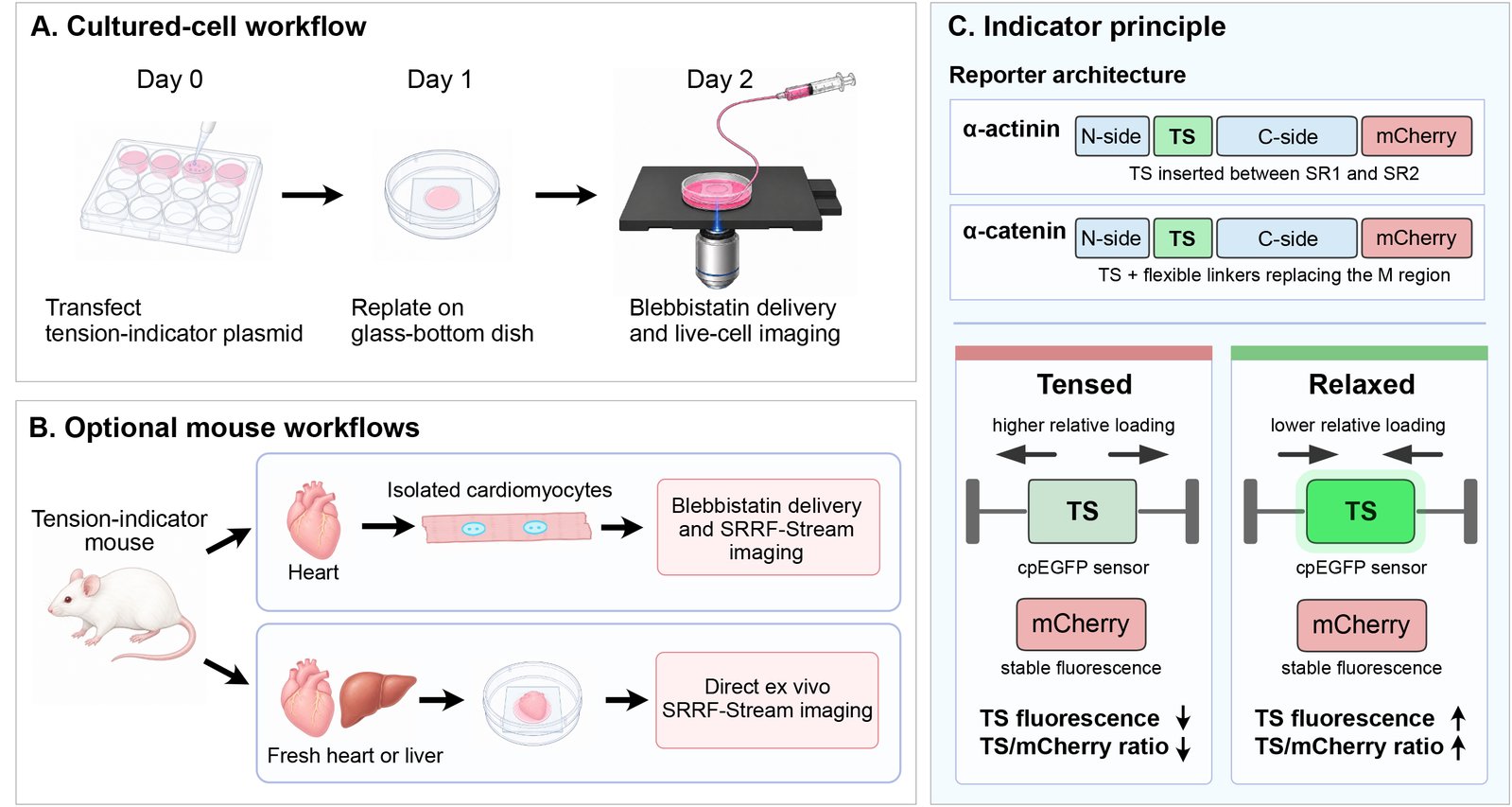


**Graphical overview of the cultured cell and optional mouse workflows.** Cultured cells are transfected on day 0, replated onto a glass-bottom dish on day 1, and subjected to blebbistatin treatment and live-cell imaging on day 2. Conventional low-magnification imaging is used primarily to quantify the blebbistatin response, while SRRF-Stream can be used for high-resolution imaging. Optional mouse workflows include blebbistatin-response SRRF-Stream imaging of isolated cardiomyocytes and direct ex vivo SRRF-Stream imaging of freshly excised heart or liver. Under matched acquisition conditions and with appropriate controls, increased molecular loading is expected to decrease TS fluorescence and the TS/mCherry ratio, whereas relaxation is expected to increase both readouts. TS denotes the tension sensor module of the indicator. Thus, relative mechanical loading is inferred from changes in the TS/mCherry ratio.

## Background

Mechanical forces regulate cell migration, tissue morphogenesis, homeostasis, and disease progression. Genetically encoded molecular tension sensors have made it possible to measure load across selected proteins in living cells. Most established sensors place a compliant linker between a donor and an acceptor fluorophore and infer extension from changes in Förster resonance energy transfer (FRET) [1–3]. These sensors are powerful, but FRET efficiency depends on both fluorophore separation and dipole orientation, and quantitative imaging generally requires bleed-through correction and carefully matched optical conditions. These constraints can become limiting in thick, optically heterogeneous tissues [3].

The indicators described here utilize a circularly permuted enhanced green fluorescent protein (cpEGFP) derived from G-CaMP8 and an internal spider-silk-derived (GPGGA)_8_ elastic linker [4–6]. Force-dependent deformation of the tension sensor (TS) module changes green fluorescence. In the α-actinin indicator, the module is inserted between spectrin repeats 1 and 2 (SR1 and SR2). In the α-catenin indicator, it replaces the central M region and is flanked by the flexible linkers GGSGGGSG and GSGCGS. The α-actinin and α-catenin indicators localize primarily to actin-rich structures and adherens junctions, respectively. Because they report different proteins in distinct molecular environments, their absolute green/red ratios should not be compared directly. In both constructs, C-terminal mCherry reports indicator abundance and serves as a reference channel. Under increasing load, TS fluorescence decreases while mCherry remains comparatively stable; under matched acquisition conditions and with appropriate controls, a higher green/red ratio is consistent with relaxation, whereas a lower ratio is consistent with higher molecular loading [4].

The method provides high spatial resolution without FRET-specific bleed-through correction and can reveal dynamic, protein-specific tension patterns in protrusions, cell margins, sarcomeres, and tissue junctions. Optical-tweezer measurements of single molecules of the purified TS module showed a reversible fluorescence response over approximately 0–6 piconewtons (pN) [4]. This range characterizes the purified TS module in single-molecule measurements but does not provide an in-cell calibration for converting each pixel’s green/red ratio into an absolute force value; higher spatial resolution therefore does not produce an absolute force map. Important limitations remain. Absolute ratios depend on expression level, optical path, detector response, and local environment; comparisons should therefore be made within the same indicator and imaging configuration. High expression levels may perturb endogenous complexes and can produce weak intermolecular FRET under artificially high transient-expression conditions, whereas no detectable intermolecular FRET was observed at physiological expression levels in cardiomyocytes [4]. For cell selection, moderate expression is defined as clearly detectable, nonsaturated signals in both channels with the expected localization and no prominent aggregates. Because TS fluorescence is relatively weak, imaging settings should be optimized to yield detectable, nonsaturated signals and kept constant across comparisons.

For selected high-resolution applications, this protocol uses SRRF-Stream reconstruction based on super-resolution radial fluctuations (SRRF), a computational method that reconstructs super-resolved images from temporal fluorescence fluctuations in a sequence of conventionally acquired images [7]. SRRF-Stream is Andor’s proprietary real-time implementation of the general SRRF algorithm in Fusion software.

## Materials and reagents

**Biological materials**

1. MDCKII cells (European Collection of Authenticated Cell Cultures, catalog number: 00062107)

2. NIH3T3 cells (ATCC, catalog number: CRL-1658)

3. pCMV_aActTS_indicator, non-FRET α-actinin tension indicator plasmid (Addgene, plasmid number: 252385)

4. pCMV_aCatTS_indicator, non-FRET α-catenin tension indicator plasmid (Addgene, plasmid number: 252386)

5. (Optional) α-actinin or α-catenin H11 knock-in tension-indicator mice crossed with Ayu-Cre mice; Slc:ICR background [4]

**Material availability:** The expression plasmids are distributed through Addgene under its applicable terms. The indicator mouse (*Mus musculus*) lines will be made available upon reasonable request to the corresponding author, subject to institutional arrangements and execution of a Material Transfer Agreement (MTA).

**Reagents**

1. Dulbecco’s modified Eagle medium (DMEM) (Thermo Fisher Scientific, catalog number: 11965092)

2. Fetal bovine serum (FBS) (Biosera, catalog number: FB-1365/500)

3. Penicillin-streptomycin-L-glutamine, 100× (Thermo Fisher Scientific, catalog number: 10378016)

4. Trypsin-EDTA (0.05%), phenol red (Thermo Fisher Scientific, catalog number: 25300054)

5. Dulbecco’s phosphate-buffered saline without calcium and magnesium [D-PBS(−)], 10× (FUJIFILM Wako, catalog number: 048-29805)

6. Ultrapure water, produced using a Milli-Q water purification system and stored in a TANKPE060 reservoir (Merck Millipore)

7. Opti-MEM I reduced serum medium (Gibco, catalog number: 31985070)

8. Lipofectamine 2000 transfection reagent (Thermo Fisher Scientific, catalog number: 11668019)

9. Gelatin (Sigma-Aldrich, catalog number: G1890-100G)

10. (−)-Blebbistatin, myosin II inhibitor (FUJIFILM Wako Pure Chemical Corporation, catalog number: 021-17041)

11. Dimethyl sulfoxide (DMSO) (Sigma-Aldrich, catalog number: D2650)

12. (Optional) Para-aminoblebbistatin, a less phototoxic/nonfluorescent blebbistatin derivative [8] (Cayman Chemical, catalog number: 22699)

13. HEPES buffer solution, 1 M (Dojindo Laboratories, catalog number: 345-06681)

14. (Optional for cardiomyocyte isolation) Heparin (Mochida Pharmaceutical, catalog number: N/A)

15. Hanks’ balanced salt solution (HBSS), 10× (Gibco, catalog number: 14185052)

16. Magnesium sulfate heptahydrate (MgSO_4_·7H_2_O) (FUJIFILM Wako, catalog number: 131-00405)

17. Taurine (FUJIFILM Wako, catalog number: 201-00112)

18. D-glucose (Sigma-Aldrich, catalog number: 07-0680)

19. 2,3-Butanedione monoxime (BDM) (Sigma-Aldrich, catalog number: B0753-25G)

20. Calcium chloride dihydrate, nuclease and protease tested (Nacalai Tesque, catalog number: 08895-15)

21. Collagenase type II (Worthington Biochemical, product code: CLS-2, catalog number: LS004176)

22. Bovine serum albumin (BSA) (FUJIFILM Wako, catalog number: 013-27054)

23. Laminin (Sigma-Aldrich, catalog number: L2020-1MG)

**Solutions**

1. Complete DMEM (see Recipes)

2. Antibiotic-free complete DMEM (see Recipes)

3. Imaging medium (see Recipes)

4. 1× D-PBS(−) (see Recipes)

5. 0.1% gelatin coating solution (see Recipes)

6. Blebbistatin stock (see Recipes)

7. Para-aminoblebbistatin stock (see Recipes)

8. Perfusion buffer (see Recipes)

9. 100 mM calcium chloride stock (see Recipes)

10. Digestion buffer (see Recipes)

11. Dissociation buffer (see Recipes)

12. Laminin coating solution (see Recipes)

**Recipes**

**1. Complete DMEM**

**Table:** 

Reagent	Final concentration	Quantity or volume
DMEM	n/a	445 mL
FBS	10% (v/v)	50 mL
Penicillin-streptomycin-L-glutamine	1×	5 mL
Total	n/a	500 mL

Prepare aseptically. Store at 4 °C.

**2. Antibiotic-free complete DMEM**

**Table:** 

Reagent	Final concentration	Quantity or volume
DMEM	n/a	450 mL
FBS	10% (v/v)	50 mL
Total	n/a	500 mL

Prepare aseptically. Store at 4 °C.

**3. Imaging medium**

**Table:** 

Reagent	Final concentration	Quantity or volume
Complete DMEM	n/a	49.5 mL
HEPES, 1 M	10 mM	500 μL
Total	n/a	50 mL

Prepare aseptically. Store at 4 °C. Equilibrate the imaging medium to room temperature before use.

**4. 1× D-PBS(−)**

**Table:** 

Reagent	Final concentration	Quantity or volume
10× D-PBS(−)	1×	100 mL
Ultrapure water	n/a	900 mL
Total	n/a	1,000 mL

Dilute 10× D-PBS(−) with ultrapure water and sterilize by autoclaving at 121 °C for 15 min. Cool to room temperature before use.

**5. 0.1% gelatin coating solution**

**Table:** 

Reagent	Final concentration	Quantity or volume
Gelatin	0.1% (w/v)	0.5 g
Ultrapure water	n/a	To 500 mL
Total	n/a	500 mL

Dissolve gelatin in ultrapure water and adjust to 500 mL. Autoclave at 121 °C for 15 min, cool to room temperature, and store at room temperature. Discard if turbidity or contamination is observed.

**6. Blebbistatin stock**

**Table:** 

Reagent	Final concentration	Quantity or volume
Blebbistatin	5 mM	1 mg
DMSO	n/a	684 μL
Total	n/a	684 μL

Dissolve blebbistatin in DMSO and aliquot the solution into microcentrifuge tubes. Wrap each tube in aluminum foil and store at -20 °C. Before use, warm an aliquot to 37 °C and mix until any precipitate has redissolved. Limit each aliquot to two freeze/thaw cycles and discard if precipitation persists.

**7. Para-aminoblebbistatin stock**

**Table:** 

Reagent	Final concentration	Quantity or volume
Para-aminoblebbistatin	12.5 mg/mL (40.67 mM)	500 μg
DMSO	n/a	40 μL
Total	n/a	40 μL

Dissolve para-aminoblebbistatin in DMSO, aliquot the solution into microcentrifuge tubes, and store at -20 °C. Thaw and mix an aliquot before use. Avoid repeated freeze/thaw cycles.

**8. Perfusion buffer (optional)**

**Table:** 

Reagent	Final concentration	Quantity or volume
HBSS 10×	1×	50 mL
MgSO_4_·7H_2_O	0.02% (w/v)	0.1 g
Taurine	0.06% (w/v)	0.3 g
D-glucose	0.08% (w/v)	0.4 g
BDM	0.1% (w/v)	0.5 g
Ultrapure water	n/a	to 500 mL
Total	n/a	500 mL

Combine 50 mL of 10× HBSS with approximately 400 mL of ultrapure water. Add MgSO_4_·7H_2_O, taurine, D-glucose, and BDM sequentially, mixing after each addition until completely dissolved. Adjust the final volume to 500 mL with ultrapure water. Prepare fresh on the day of cardiomyocyte isolation and discard any unused buffer.

**9. 100 mM calcium chloride stock (optional)**

**Table:** 

Reagent	Final concentration	Quantity or volume
Calcium chloride dihydrate	100 mM	147.0 mg
Ultrapure water	n/a	to 10 mL
Total	n/a	10 mL

Dissolve 147.0 mg of calcium chloride dihydrate in approximately 8 mL of ultrapure water. Adjust the final volume to 10 mL with ultrapure water. Sterilize the solution using a 0.20-μm syringe filter and store at 4 °C.

**10. Digestion buffer (optional)**

**Table:** 

Reagent	Final concentration	Quantity or volume
Collagenase II	1 mg/mL	40 mg
100 mM CaCl_2_ stock	100 μM	40 μL
Perfusion buffer	n/a	to 40 mL
Total	n/a	40 mL

Dissolve collagenase II in the perfusion buffer, add 40 μL of the 100 mM CaCl_2_ stock, and adjust the final volume to 40 mL with perfusion buffer. Prepare immediately before use and maintain at 37 °C until perfusion.

**11. Dissociation buffer (optional)**

**Table:** 

Reagent	Final concentration	Quantity or volume
BSA	4% (w/v)	1.2 g
Perfusion buffer	n/a	to 30 mL
Total	n/a	30 mL

Dissolve BSA in the perfusion buffer and adjust the final volume to 30 mL. Keep at room temperature and use on the day of cardiomyocyte isolation. This BSA-containing buffer is used to terminate collagenase digestion, dissociate the heart tissue, and plate isolated cardiomyocytes.

**12. Laminin coating solution (optional)**

**Table:** 

Reagent	Final concentration	Quantity or volume
Laminin (1–2 mg/mL)	1:100 dilution; 10–20 μg/mL	50 μL
1× D-PBS(−)	n/a	4.95 mL
Total	n/a	5 mL

Dilute the laminin stock (1–2 mg/mL, as specified on the supplier’s label) 1:100 in sterile 1× D-PBS(−) to obtain a working concentration of 10–20 μg/mL. Incubate the glass-bottom dish with the diluted laminin at 37 °C for 1 h.

**Laboratory supplies**

1. 35-mm glass-bottom dishes with a 14-mm diameter No. 1S glass-bottom area (Matsunami Glass, catalog number: D11131H)

2. 60-mm tissue-culture dishes (Thermo Fisher Scientific, catalog number: 150462)

3. 12-well tissue-culture plates (Thermo Fisher Scientific, catalog number: 150628)

4. 200- and 1,000-μL pipette tips (WATSON and SHS-TIP, respectively, or equivalent)

5. Sterile serological pipettes, 5, 10, and 25 mL (VIOLAMO, Thermo Fisher Scientific, or equivalent)

6. Sterile polypropylene conical centrifuge tubes, 15 and 50 mL (Thermo Scientific Nunc, catalog numbers: 339650 and 339652)

7. 1.5-mL polypropylene microcentrifuge tubes (Eppendorf or equivalent) and aluminum foil for protecting blebbistatin solutions from light

8. Modified 35-mm dish lid with an opening, flexible delivery tubing, three-way stopcock, syringe, and adhesive tape for securing the dish and tubing during blebbistatin delivery

9. (Optional for cardiomyocyte isolation) Blunt-end 18 G cannula, 22 G needle, and 20-mL syringe

10. (Optional for cardiomyocyte isolation) Three-way stopcocks, tubing compatible with the peristaltic pump, rubber stopper, and insulating material

11. (Optional for cardiomyocyte isolation) Rounded-tip glass pipettes with progressively smaller bore diameters

12. (Optional for cardiomyocyte isolation) 4-0 PERMA-HAND silk suture (ETHICON, Johnson & Johnson, catalog number: 1687G)

13. (Optional for cardiomyocyte isolation) NB 40 nylon mesh with a 435-μm opening (NBC Meshtec or equivalent)

14. (Optional for cardiomyocyte isolation) Sterile 25-mm syringe filter with a 0.20-μm mixed cellulose ester (MCE) membrane (ADVANTEC, catalog number: 25AS020AS)

15. (Optional for cardiomyocyte isolation and organ collection) Dissection scissors and forceps

16. (Optional for organ imaging) Clean plastic wrap for immobilizing freshly isolated organs (Saran Wrap or equivalent)

## Equipment

1. Precision electronic balance (VIBRA/Shinko Denshi Co., Ltd., model: AJII-3200; readability: 0.01 g)

2. Analytical balance (METTLER TOLEDO, model: ME54T; readability: 0.1 mg)

3. Laboratory autoclave (TOMY SEIKO Co., Ltd., model: LSX-500)

4. Adjustable micropipettes covering 2–1,000 μL (Gilson or equivalent)

5. Electric pipette controller (Pipet-Aid XP, Drummond Scientific, catalog number: 4-000-101)

6. Clean bench (S-1300P, Showa Science Co., Ltd.)

7. Humidified direct-heat CO_2_ incubator (Thermo Scientific, Forma Model 310), set to 37 °C and 5% CO_2_

8. Tabletop centrifuge compatible with 15- and 50-mL conical tubes (KUBOTA Corporation, model: 4000)

9. Constant-temperature water bath (NTT-2300 immersion thermostat with SB-9 bath, EYELA/Tokyo Rikakikai Co., Ltd.), set to 37 °C for buffer warming or 45 °C for circulation through the water jacket of the Langendorff reservoir, as specified in the procedure

10. Inverted phase contrast microscope (Olympus, model: CKX31)

11. Bürker–Türk hemocytometer with a 0.1-mm chamber depth (ERMA, Tokyo, Japan)

12. Vortex mixer (Scientific Industries, model: Vortex-Genie 2)

13. Nikon Eclipse Ti microscope with C1 confocal scanner, Perfect Focus System, Plan Apo 20×/0.75 NA and Plan Apo 40×/0.95 NA objectives, 405-, 488-, and 561-nm lasers (for multipoint time-lapse imaging)

14. Olympus FV3000 confocal laser-scanning microscope with UPLSAPO40X2/0.95 NA, PLAPON60XOSC2/1.4 NA oil, and UPLSAPO100XO/1.4 NA oil objectives (for standard confocal imaging)

15. Dragonfly 500 spinning-disk confocal system (Andor Technology/Oxford Instruments) on an Olympus IX83 base with Zyla 4.2 Plus sCMOS and iXon Life 888 EMCCD cameras, UPLXAPO20X/0.4 NA, UPLXAPO60XO/1.42 NA oil, UPLSAPO60XS2/1.30 NA silicone-immersion objective, and UPLXAPO100XO/1.45 NA oil objectives (for dual-color, SRRF-Stream, and direct organ imaging)

16. SIL300CS-30CC silicone immersion oil (Evident, catalog number: N4190800)

17. (Optional for cardiomyocyte isolation) Water-jacketed glass reservoir containing a coiled glass perfusate tube, with an approximately 20-mL buffer capacity (manufacturer and model unknown)

18. (Optional for cardiomyocyte isolation) Peristaltic tube pump (EYELA/Tokyo Rikakikai Co., Ltd., model: SMP-23) for recirculation of digestion buffer during Langendorff perfusion

## Software and datasets

1. Fiji (ImageJ2 version 2.16.0; ImageJ1 version 1.54p; free and open source) for image processing, region-of-interest selection, fluorescence quantification, and ratiometric image generation [9]

2. Chromagnon (free and open source) for chromatic-shift correction of two-color images [10]

3. Fusion software with SRRF-Stream functionality (Andor Technology/Oxford Instruments; proprietary software supplied with the Dragonfly system) for Dragonfly image acquisition and SRRF-Stream reconstruction

4. EZ-C1 (Nikon; proprietary software supplied with the Nikon C1 system) for Nikon C1 acquisition

5. FLUOVIEW FV3000 (Olympus; proprietary software supplied with the FV3000 system) for FV3000 acquisition

6. Imaris 10.2 (Oxford Instruments; commercial license required; optional) was used only to generate the representative 3D reconstruction and is not part of the core workflow. It is not required for image preparation, ROI measurement, ratio analysis, or statistical analysis

7. Python 3.11.12 with pandas 2.3.3 and NumPy 1.26.4, was used to organize measurements exported from Fiji; SciPy 1.15.2 was used for statistical testing; and Matplotlib 3.10.3 and Seaborn 0.13.2 was used for plot and heatmap generation, as described in the quantitative-analysis section. Fiji was used for image preparation and ROI measurement. Equivalent statistical and plotting software may be substituted

8. Published datasets and representative movies associated with Fujiwara et al. [4] are available in Supplementary Information: numerical source data (Supplementary Data 1), plasmid DNA sequences (Supplementary Data 2), and time-lapse movies of cultured cells (Supplementary Movies 1–4) and isolated cardiomyocytes (Supplementary Movies 5 and 6)

*Note: The versions of the instrument-control software varied between experiments because the acquisition workstations were updated during the study. The protocol does not depend on a specific software version.*

## Procedure

The core cultured-cell workflow requires approximately two days after transfection: transfect cells on day 0, replate transfected cells on the glass-bottom dish on day 1, and image on day 2. Perform all cell-culture manipulations aseptically.

**A. Prepare cells and coated imaging dishes**

1. Maintain MDCKII or NIH3T3 cells in complete DMEM in 60-mm tissue-culture dishes at 37 °C in a humidified incubator with 5% CO_2_.

2. Passage the cells before they reach confluence and use cultures in the logarithmic growth phase. Use cells with normal morphology, maintain a consistent passage range across comparisons, and confirm mycoplasma-negative status before use.

3. Add 500 μL of 0.1% gelatin coating solution to each well of a 12-well plate and 200 μL to the glass-bottom area of each 35-mm glass-bottom dish. Incubate for approximately 30 min.

4. Aspirate the gelatin solution and allow the coated surfaces to air-dry in a clean bench for approximately 5 min, until no visible liquid remains. Prolonged desiccation is not intended. Prepare the coated vessels immediately before use.

**B. Transfect the α-actinin or α-catenin tension indicator and replate cells for imaging**

1. On the day before transfection (day -1), seed either MDCKII or NIH3T3 cells at 1.0 × 10^5^ cells per well in gelatin-coated 12-well plates using 1 mL of antibiotic-free complete DMEM per well.

**Critical:** Do not swirl or rock the plate after seeding, as this causes cells to accumulate in the center of the wells. Carefully return the plate to the incubator.

2. On day 0, confirm that the cells are approximately 80% confluent and prepare the DNA–Lipofectamine 2000 complexes for each well as follows:

a. Dilute 2 μg of pCMV_aActTS_indicator or pCMV_aCatTS_indicator in 100 μL of Opti-MEM.

b. In a separate microcentrifuge tube, dilute 4 μL of Lipofectamine 2000 in 100 μL of Opti-MEM.

c. Incubate both mixtures separately for 5 min at room temperature.

d. Combine the DNA and Lipofectamine mixtures and vortex for approximately 5 s at speed setting 3–4 using a Vortex-Genie 2. Incubate the combined mixture for 20 min at room temperature.

**Critical:** Avoid prolonged or vigorous vortexing after combining the DNA and Lipofectamine mixtures.

e. Add the entire DNA–Lipofectamine complex dropwise to one well containing 1 mL of antibiotic-free DMEM. Carefully return the plate to the incubator without disturbing it.

**Critical:** Do not swirl or rock the plate after adding the DNA–Lipofectamine complex.

3. Incubate the transfected cells overnight at 37 °C in a humidified incubator with 5% CO_2_.

4. Approximately 24 h after transfection (day 1), aspirate the culture medium. Rinse the cells twice with 1 mL of 1× D-PBS(−) per well, aspirating the D-PBS(−) completely after each wash.

5. Add 200 μL of 0.05% trypsin-EDTA to each well and incubate for 5 min at 37 °C in a humidified incubator with 5% CO_2_.

6. Confirm cell detachment using an inverted phase contrast microscope. Add 2 mL of complete DMEM to neutralize the trypsin and gently pipette to obtain a single-cell suspension.

7. Transfer the cell suspension to a 15-mL conical tube and centrifuge at 190× *g* for 3 min at room temperature.

8. Carefully remove the supernatant and resuspend the cell pellet in 1 mL of complete DMEM.

9. Count the cells using a Bürker–Türk hemocytometer and adjust the cell density as follows: NIH3T3 cells, 1.0 × 10^5^ cells/mL; MDCKII cells, 2.0 × 10^5^ cells/mL.

10. Carefully dispense 200 μL of the cell suspension onto the center of the gelatin-coated glass-bottom area of each 35-mm dish. This corresponds to 2.0 × 10^4^ NIH3T3 cells or 4.0 × 10^4^ MDCKII cells per dish.

**Critical:** Keep the suspension confined to the glass-bottom area. Do not swirl or rock the dish after plating; carefully transfer it to the incubator to prevent the cells from accumulating in the center.

11. Incubate the cells overnight at 37 °C in a humidified incubator with 5% CO_2_.

12. On day 2, without removing the initial 200 μL of medium, carefully add 2 mL of prewarmed complete DMEM and maintain the cells until imaging, which is started approximately 16–20 h after replating.

**Critical:** At the time of imaging, cultures should be no more than approximately 80% confluent. Do not use cultures approaching 90% confluence.

*Note: The apparent transfection efficiency is approximately 5%–10% based on visual inspection, although it was not formally quantified. No numerical viability threshold was used; exclude cultures showing widespread cell rounding or detachment.*

**C. Configure two-color live-cell imaging**

1. Perform live-cell imaging in a room set to approximately 25 °C, without stage temperature control, stage thermometry, or external CO_2_ supply. Allow the medium-filled dish to equilibrate until focus is stable, record the room temperature for each experiment, and keep the timing consistent across directly compared conditions.

*Note: Temperature equilibration is important because changes in dish temperature can cause substantial focus drift. In drug-treatment experiments, half of the medium is exchanged to achieve the specified final drug concentration. In our setup, performing this exchange at 37 °C caused thermal drift, while manipulation within a closed CO_2_-controlled chamber was prone to positional shifts. Room-temperature imaging without external CO_2_ was therefore used to improve imaging stability. HEPES-buffered medium helps limit pH changes, but these nonphysiological conditions may affect cell physiology or drug responses; imaging should therefore be kept brief and time-matched across directly compared conditions.*

2. Locate transfected cells using the lowest practical illumination. Confirm that both TS and mCherry are detectable and that the indicator shows the expected localization.

3. Acquire the TS channel with 488-nm excitation and the mCherry reference channel with 561-nm excitation. Use the filter sets in Table 1 or equivalent nonoverlapping emission windows. Images were acquired in a single focal plane at each time point. The TS and mCherry channels were acquired sequentially, with the TS channel acquired first. The interchannel delay was controlled by the acquisition software and was not recorded. Use a 512 × 512-pixel acquisition matrix for Nikon C1 imaging. For Dragonfly 500 imaging, image dimensions depend on the acquisition mode; the retained representative conventional dataset was 2,048 × 2,048 pixels, whereas the representative SRRF-Stream dataset described in Section E produced 4,096 × 4,096-pixel reconstructed images. Use the minimum pinhole setting available on the confocal system. Select laser power, detector gain, and exposure or dwell time to obtain detectable, nonsaturated signals. Keep physical pixel size, pinhole, and all other acquisition settings unchanged within each directly compared dataset.

**Table 1.** Imaging channels and acquisition order used in the original study. Equivalent filters may be used, but the same configuration must be retained within an experiment.

System	Channel	Acquisition order	Excitation	Emission
Nikon C1	TS	1st	488 nm	525/50 nm
Nikon C1	mCherry	2nd	561 nm	595/40 nm
Dragonfly 500	TS	1st	488 nm	521/38 nm
Dragonfly 500	mCherry	2nd	561 nm	594/43 nm

**Critical:** Keep all acquisition settings unchanged across time points and directly compared conditions and avoid saturation in either channel. For quantitative analysis, exclude ROIs containing any saturated pixels in either channel and time series showing visible focus drift that prevents analysis of the same structure throughout the acquisition.

**D. Validate indicator responsiveness with blebbistatin**

1. Immediately before imaging, replace the culture medium with 1 mL of imaging medium equilibrated to room temperature.

*Notes:*

*1. The HEPES-containing imaging medium limits pH fluctuations during short-term imaging without an external CO_2_ supply.*

*2. Phenol red may increase fluorescence background and light absorption; if background is limiting, use an otherwise matched phenol-red-free imaging medium.*

2. Immediately before imaging, add 3 μL of the 5 mM blebbistatin stock to 497 μL of imaging medium equilibrated to room temperature to prepare a 30 μM delivery solution.

*Note: The delivery solution is diluted 1:1 with the 500 μL of medium remaining in the dish, resulting in a final blebbistatin concentration of 15 μM.*

3. For the dose–response experiment, prepare blebbistatin delivery solutions to yield final concentrations of 0, 5, 10, 15, and 20 μM after the 1:1 medium exchange, as shown in Table 2. For the 0 μM condition, use imaging medium without blebbistatin or added DMSO. The original dose–response experiment was performed without matching the DMSO concentration across conditions; this is a limitation of the original design. A DMSO-matched vehicle design is recommended for future experiments.

**Table 2.** Blebbistatin delivery solutions used in the original dose–response experiment

Final concentration in the dish	5 mM blebbistatin stock	Imaging medium
0 μM	0 μL	500 μL
5 μM	1 μL	499 μL
10 μM	2 μL	498 μL
15 μM	3 μL	497 μL
20 μM	4 μL	496 μL

4. Cover the dish with a modified lid containing a hole for the delivery tubing. Pass the tubing through the hole. Before use, fill the tubing completely with the assigned delivery solution and expel any air. Position its tip in the medium near the dish wall and away from the selected imaging fields. Secure the dish and tubing to the microscope stage with tape. Connect the other end of the tubing to a three-way stopcock and then to the syringe containing the blebbistatin solution.

**Critical:** Secure the dish and tubing firmly and ensure that no air bubbles are present in the delivery line.

5. Locate transfected cells using the lowest practical illumination. Confirm that both TS and mCherry are detectable and show the expected localization. Select the fields for multipoint imaging and acquire one pretreatment image (“Pre”) at each field. Complete field selection before treatment and retain all technically analyzable fields regardless of their subsequent response.

6. Without changing the field or focus, carefully remove 500 μL of medium from the dish, leaving 500 μL. Slowly deliver 500 μL of the blebbistatin solution through the tubing. Confirm delivery of 500 μL from the syringe graduations. The delivery rate and time required for complete mixing were not recorded in the original experiments; use a consistent delivery rate and record the delivery duration in new experiments.

**Critical:** Keep the dish stationary and minimize the introduction of air bubbles during medium exchange.

7. Immediately after blebbistatin delivery, begin multipoint time-lapse imaging using the Nikon C1 system. Define the first post-treatment image acquired at each field as t = 0 min and subsequently acquire images at 1-min intervals through t = 5 min, giving six post-treatment images and seven images in total, including Pre. The exact delays between the Pre image, drug delivery, and first post-treatment image were not recorded in the original experiments. Record these times in new experiments.

8. Analyze all preselected, technically analyzable fields as described in Data analysis. Do not exclude a field because it fails to show the expected increase in TS fluorescence.

**Caution:** Blebbistatin is light-sensitive and can be phototoxic during blue-light excitation. Protect stocks and working solutions from ambient light, minimize 488-nm illumination, and consider para-aminoblebbistatin when prolonged imaging is required [8] or as an independent confirmation in new validation experiments.

**Quality control:** For new experiments, include a DMSO-matched vehicle control and cell-free background ROIs in both channels. Do not attribute a green-channel increase to the sensor if it is also present in cell-free regions or accompanied by visible precipitates.

**Expected result:** Blebbistatin-mediated relaxation produces an increase in TS fluorescence and in the relaxation ratio. This expected response is not an exclusion criterion, and no numerical response-based cutoff is applied.

*Note: In Figure 1, cell-free regions were excluded during ROI selection, but no separate background subtraction was applied.*

Representative results are shown in Figure 1.

**Figure 1.**
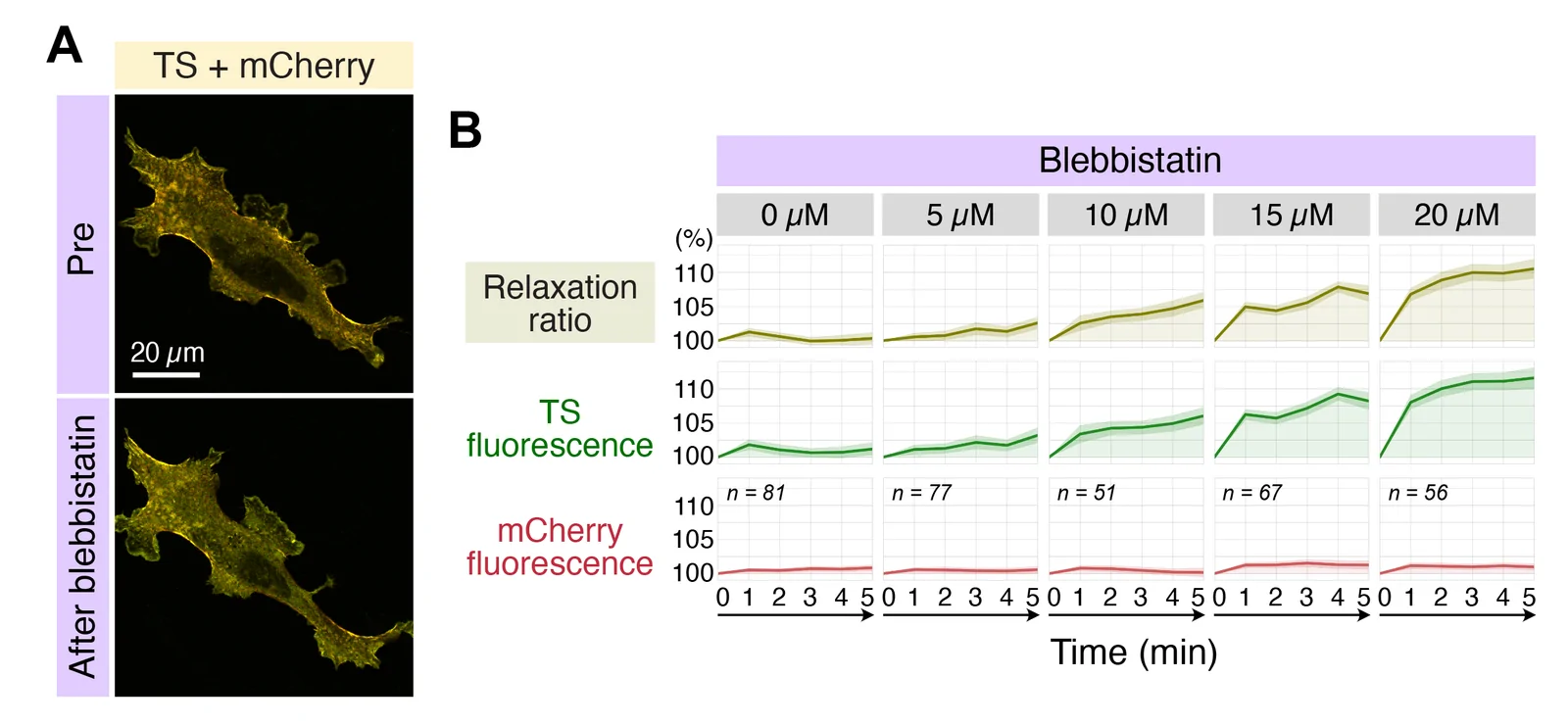
Representative response and dose-dependent validation of the α-actinin tension indicator following blebbistatin treatment. (A) Representative merged TS and mCherry images of an NIH3T3 cell before (Pre) and after blebbistatin treatment. Scale bar, 20 μm. (B) Time courses of the normalized relaxation ratio, TS fluorescence, and mCherry fluorescence in MDCKII cells treated with 0, 5, 10, 15, or 20 μM blebbistatin. Values were normalized to those at 0 min. Lines and shaded areas indicate the mean and 95% confidence intervals, respectively. The number of analyzed cells is indicated for each concentration. TS, tension sensor module. Adapted from Figure 1E, F of Fujiwara et al. [4] under the Creative Commons Attribution 4.0 International License.

**E. Image subcellular tension dynamics at high resolution**

1. For lamellipodia and filopodia, select an NIH3T3 cell expressing the α-actinin tension indicator with well-resolved protrusions. For adherens-junction imaging, select an NIH3T3 cell expressing the α-catenin tension indicator with a clearly resolved cell margin or protrusion.

2. Use a high-numerical-aperture oil-immersion objective appropriate to the field of view; 60×/1.42 NA and 100×/1.45 NA objectives were used in the original datasets.

3. For imaging of the α-actinin tension indicator in lamellipodia and filopodia, acquire standard dual-color time-lapse images without SRRF-Stream reconstruction. For imaging of the relatively weak α-catenin tension-indicator signal at cell margins and protrusions, use SRRF-Stream imaging. Acquire 20 raw frames per channel per reconstructed time point using the iXon Life 888 EMCCD camera and reconstruct the images in Fusion 2.2.0.50 software. Twenty frames rather than the default 100 were used to limit photobleaching. Set radiality magnification to 4, ring radius to 2, temporal analysis to “Mean,” and fixed-pattern-noise correction to off. Use identical reconstruction settings for both channels. In the representative dataset, 1,024 × 1,024-pixel input frames produced 4,096 × 4,096-pixel reconstructed images. Both channels were acquired with a 50-ms exposure and EM gain 300; for other images, acquisition settings were adjusted to the objective and signal level and kept unchanged within each direct comparison. The representative time series was acquired at 1-min intervals.

4. Correct chromatic shift between the TS and mCherry channels using Chromagnon and a reference image acquired with the same objective and optical configuration [10]. Use a single-plane, two-channel reference image containing a corresponding junctional or cytoskeletal structure. Set TS as the reference channel, apply the default two-dimensional translation, rotation, and x/y magnification correction, save the transform as a CSV file, and apply it to the corresponding images. Accept the registration when no visible displacement remains; no bead reference or numerical residual-error threshold was used.

5. Define line or region-of-interest trajectories along the leading edge, filopodium, cell protrusion, or cell margin. Retain the same trajectory orientation across time.

6. Generate green/red ratio images and position-by-time heatmaps as described in Data analysis. Use conventional images for primary ratio quantification; use SRRF-Stream-derived ratios quantitatively only after confirming agreement with conventional ratios over the same ROI.

**F. (Optional) Isolate and image cardiomyocytes from α-actinin tension-indicator mice**

1. Add 200 μL of the 1:100 laminin coating solution to the glass-bottom area of a 35-mm dish. Incubate for 1 h at 37 °C in a humidified incubator, aspirate the solution, and allow the surface to air-dry.

2. Set up the recirculating Langendorff perfusion circuit shown in Figure 2. Connect the lower and upper ports of the outer water jacket to a circulating water bath set to 45 °C, so that water enters through the lower port and exits through the upper port. Close the upper opening of the glass reservoir with a rubber stopper. Insert a 22 G needle through the stopper and attach a three-way stopcock (upper stopcock) to the external end of the needle. Load the pump tubing into the peristaltic pump, connect one end to the upper stopcock, and place the other end in a sterile 50-mL conical tube beneath the heart. Set the pump direction so that the digestion buffer is drawn from the conical tube and delivered through the upper stopcock and 22 G needle into the glass reservoir.

**Figure 2.**
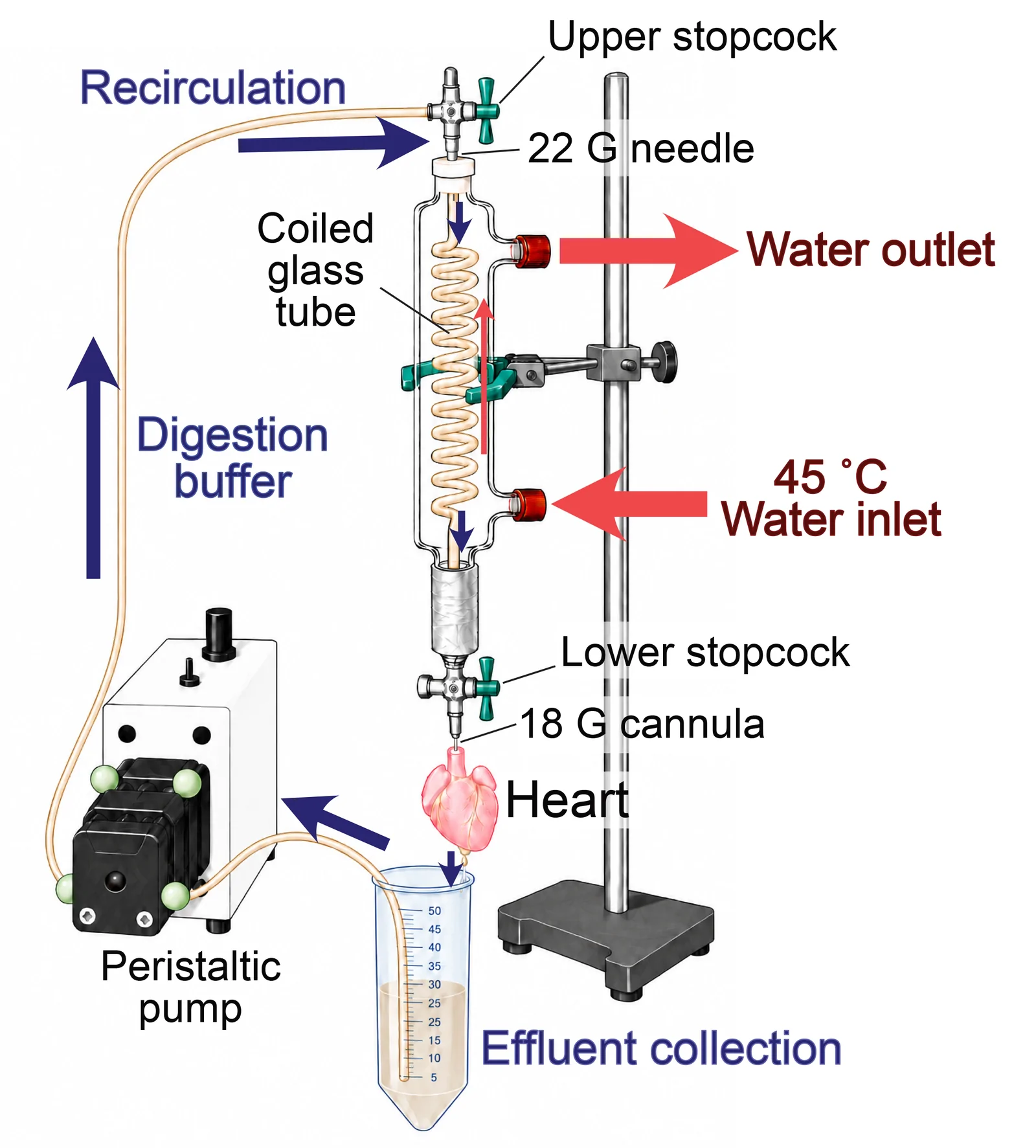
Recirculating Langendorff perfusion circuit for cardiomyocyte isolation. During collagenase digestion, the digestion buffer flows downward through the coiled glass tube and perfuses the heart through the lower three-way stopcock and 18-G cannula. The effluent drains into a sterile 50-mL conical tube and is returned through the pump tubing, upper three-way stopcock, and 22-G needle to the upper inlet of the glass reservoir by the SMP-23 peristaltic pump. Water from a circulating bath set to 45 °C enters the lower port of the outer water jacket and exits through the upper port, warming the digestion buffer as it passes through the coiled glass tube. Blue arrows indicate digestion-buffer flow, and red arrows indicate circulating-water flow. The initial flushing of the heart with 15 mL of perfusion buffer using a syringe is not shown.

3. Connect the lower outlet of the coiled glass tube through insulated tubing to a second three-way stopcock (lower stopcock). Prime the pump tubing and coiled glass tube with digestion buffer and remove all air bubbles.

**Critical:** Ensure that the perfusion path downstream of the coiled glass tube is filled with digestion buffer and free of air before connecting the heart. The perfusate temperature measured at the cannula outlet was 38–39 °C.

*Note: Wrap the tubing between the glass reservoir and the heart with insulating material. The water bath is set to 45 °C to compensate for heat loss along this external flow path; this setting does not represent the temperature of the buffer delivered to the heart.*

4. Inject a 4–6-week-old male or female indicator mouse weighing approximately 35–40 g intraperitoneally with 150 μL of heparin containing 150 IU. This fixed dose was used for all mice within this age and weight range.

5. After 5 min, euthanize the mouse by cervical dislocation in accordance with the approved animal protocol.

6. Rapidly excise the heart and cannulate the ascending aorta with a blunt-end 18 G cannula. Secure the aorta around the cannula with a 4-0 silk suture tied in a double knot. Connect the cannula to a 20-mL syringe and gently perfuse the heart with 15 mL of perfusion buffer to remove the blood.

*Note: BDM in the perfusion and digestion buffers suppresses cardiomyocyte contraction and helps limit hypercontracture-associated cell damage during isolation.*

7. Start the digestion-buffer flow and allow the buffer to drip from the lower stopcock. Leaving the cannula secured in the aorta, disconnect the syringe, fill the cannula hub with digestion buffer, and connect it to the lower stopcock without trapping air.

**Critical:** Do not allow air to enter the cannula when switching from perfusion buffer to digestion buffer.

8. Perfuse the heart with digestion buffer for 12 min. Collect the effluent in the 50-mL conical tube and recirculate it to the upper inlet of the glass reservoir using the SMP-23 peristaltic pump at a measured flow rate of approximately 2.8 mL/min (dial setting 3). Perfusion pressure and a formal flow-acceptance range were not established. Digestion was stopped after 12 min, when the tissue could be dispersed by gentle trituration. The relatively high perfusate temperature may shorten the required digestion time.

9. Transfer the digested heart to 4 mL of dissociation buffer. Triturate gently, beginning with a rounded-tip, large-bore glass pipette and progressing to narrower pipettes until the readily dissociable tissue has dispersed and the remaining tissue fragments no longer dissociate with gentle trituration.

**Critical:** Reduce the pipette bore gradually and avoid vigorous trituration. The pipette bore sizes and number of strokes were not standardized. Stop trituration when the remaining tissue fragments no longer dissociate readily; do not continue solely to eliminate all visible fragments.

10. Pass the cell suspension through an NB 40 (OP435) nylon mesh with a 435-μm opening into a sterile 50-mL conical tube.

11. Centrifuge at 20× *g* for 1 min at room temperature. Carefully remove the supernatant.

12. Gently resuspend the cardiomyocytes in 1 mL of dissociation buffer and divide the suspension equally among four laminin-coated dishes. Incubate for 1 h at 37 °C and 5% CO_2_ to allow attachment. Transfer the dish to the microscope and perform imaging at room temperature without an external CO_2_ supply, while keeping the cardiomyocytes in the BDM-containing dissociation buffer. Allow the dish to reach thermal equilibrium before acquiring the pretreatment image. Select intact, rod-shaped cardiomyocytes with organized sarcomeres and exclude rounded or visibly damaged cells.

*Note: BDM is intentionally maintained throughout imaging to suppress spontaneous cardiomyocyte contraction and minimize motion artifacts. The entire 1-mL suspension from one heart was plated in four dishes. Yield and viability were not formally quantified; a preparation was considered suitable when attached, rod-shaped cardiomyocytes with organized sarcomeres were available for imaging.*

13. Using the Dragonfly 500 system and iXon Life 888 EMCCD camera, acquire one pretreatment SRRF-Stream image (“Pre”) of the TS and mCherry channels using 20 raw frames per reconstructed time point.

14. Immediately before treatment, prepare 500 μL of 20 μM blebbistatin delivery solution by mixing 2 μL of the 5 mM stock with 498 μL of dissociation buffer. Remove 500 μL of dissociation buffer from the dish and replace it with the delivery solution, giving a final blebbistatin concentration of 10 μM while maintaining the BDM concentration. For the BDM-only control, perform the same medium exchange using BDM-containing dissociation buffer without blebbistatin. This control produced no detectable change in the TS/mCherry ratio.

**Critical:** Keep the dish stationary and minimize air-bubble formation during medium exchange.

15. Acquire the first post-treatment image immediately after blebbistatin delivery and define it as t = 0 min. Continue SRRF-Stream imaging at 1-min intervals for up to 30 min, using identical acquisition and reconstruction parameters throughout. Because BDM is present throughout imaging, the blebbistatin response represents additional relaxation beyond the BDM-treated state. Use Pre as the primary baseline for the immediate BDM/blebbistatin comparison; use normalization to t = 0 only for a separate description of post-delivery kinetics.

**G. (Optional) Direct ex vivo SRRF-Stream imaging of freshly isolated organs**

1. Following euthanasia in accordance with the approved animal protocol, rapidly excise the heart or liver from an α-actinin or α-catenin tension-indicator mouse.

2. Prepare the organ as follows:

a. Liver: Gently rinse the liver with 1× D-PBS(−) to remove visible surface blood.

b. Heart: Cannulate the ascending aorta and perfuse the heart with 15 mL of perfusion buffer to remove the blood and arrest beating, as described in Section F.

3. Place the organ in 1× D-PBS(−) in a 35-mm glass-bottom dish, with the left ventricular epicardial surface of the heart or the ventral surface of the liver in direct contact with the glass-bottom area.

4. Loosely place, without stretching, a small piece of clean plastic wrap to immobilize it and prevent drying.

**Critical:** Confirm at low magnification that application of the plastic wrap does not alter the gross tissue contour or produce visible indentation and that the surface of interest remains in contact with the glass.

5. Immediately place the dish on the Dragonfly 500 spinning-disk confocal system and perform imaging at room temperature without an external CO_2_ supply. Locate the tissue surface using the UPLSAPO60XS2/1.30 NA silicone-immersion objective with SIL300CS-30CC silicone immersion oil and the lowest practical illumination. Focus on the superficial tissue plane directly adjacent to the glass and complete imaging within 30 min of euthanasia.

6. Acquire the TS and mCherry channels using the iXon Life 888 EMCCD camera. Acquire 20 raw frames per channel for each SRRF-Stream image and reconstruct the images using Fusion software.

**Critical:** Use identical acquisition and SRRF-Stream reconstruction settings for all samples or regions being directly compared and avoid saturation in either channel.

7. Correct chromatic shift between the TS and mCherry channels and generate ratiometric images as described in the Data analysis section.

## Data analysis

**A. Image preparation and quality control**

1. Open the green TS and red mCherry image series in Fiji. Preserve the raw data and perform calculations on duplicate image stacks.

2. For SRRF-Stream or other high-resolution two-color images, apply the predetermined chromatic-shift correction before calculating ratios.

3. Inspect each field for saturation, focus drift, stage movement, cell detachment, phototoxic morphology, and loss of the red reference signal.

4. Include only cells or regions that show detectable, nonsaturated signal in both channels and the expected subcellular localization. For 16-bit images, exclude an ROI if any pixel reaches the digital maximum of 65,535; for new quantitative analyses, require the mean ROI signal to exceed the mean local background in both channels. Exclude dead or detaching cells, frames with unrecoverable focus/stage jumps, saturated regions, and regions in which the reference signal becomes unreliable. The original analysis did not use prespecified numerical thresholds for focus drift, residual registration error, or mCherry loss; these thresholds should therefore be defined prospectively for new experiments rather than reconstructed retrospectively.

**Original processing:** The published pixel-wise ratio maps were generated without masking, intensity gating, or weighting by mCherry signal [4] and were used for qualitative visualization. TS and mCherry are components of the same fusion protein; therefore, TS-positive structures also contain an mCherry signal. Quantitative conclusions were based on ROI-level green/red ratios measured using identical ROIs in the two registered channels, not on individual pixel values.

**B. Region-of-interest fluorescence analysis**

1. Draw an ROI around each cell or intracellular structure and measure the integrated intensity (IntDen) separately in the green and red channels at every time point. Manually redraw the ROI at each time point to follow the same cell or structure and match its outline as closely as possible, and apply the identical ROI to both channels. Cell position and morphology generally changed only minimally during the 5-min time course. The original ROI selection was not formally blinded; anonymized file names are recommended for future group comparisons. The original published analysis used raw integrated density (IntDen) without background subtraction. Background subtraction was examined but did not materially alter the observed pattern; raw IntDen was therefore retained to minimize additional processing. Accordingly, *I_C_(t*) denotes raw IntDen throughout this protocol.

2. For each channel *C*, normalize the selected intensity measure *I_C_* to its value at the first analyzed frame:

$$F_{C}\left(t\right)=100\times I_{C}\left(t\right)/I_{C}\left(0\right)$$

This yields *F_G_(t*) for TS and *F_R_(t*) for mCherry, each expressed as a percentage of its initial value.

3. Calculate the green-to-red ratio using the same intensity measure for both channels:

$$R_{GR}\left(t\right)=I_{G}\left(t\right)/I_{R}\left(t\right)$$

4. Normalize the green-to-red ratio to its initial value to obtain the relaxation ratio:

$$Relaxation\;ratio\left(t\right)=100\times R_{GR}\left(t\right)/R_{GR}\left(0\right)$$

A value above 100 is consistent with relaxation relative to the initial frame, whereas a value below 100 is consistent with increased molecular loading. This is a relative readout and should not be interpreted as an absolute force measurement.

5. To reproduce the local SRRF-Stream analysis used in the original study, the difference between the independently normalized channels may also be calculated as follows:

$$\Delta_{GR}\left(t\right)=F_{G}\left(t\right)-F_{R}\left(t\right)$$

A positive Δ*_GR_* indicates that TS fluorescence increased relative to mCherry. This zero-centered metric was used for selected SRRF-Stream analyses because it makes small divergences between the independently normalized channels more readily visible than the ratio. The green/red ratio is the primary readout for conventional ROI analyses, whereas Δ*_GR_* is used only for selected SRRF-Stream visualizations.

*Note: For most blebbistatin-delivery analyses in the original study, the first post-delivery frame was defined as t = 0 min and set to 100% for normalization. The pretreatment image (Pre) was analyzed separately because medium exchange could cause transient temperature-dependent focus drift or positional shift. Thus, these analyses describe changes after the first post-delivery frame and do not quantify the immediate Pre-to-post response. For the cardiomyocyte BDM/blebbistatin workflow in Section F, use Pre as the primary baseline and report the first post-delivery frame separately.*

**C. Pixel-wise ratio maps and position-by-time heatmaps**

1. Divide the green image by the corresponding red image pixel by pixel to create a green/red ratio image for each time point. Use the pixel-wise maps and derived position-by-time heatmaps for qualitative visualization; base quantitative comparisons on the ROI-level ratios described in Section B.

2. Render the ratio image using the “jet” colormap. In the convention used in the original study, warm colors indicate higher ratios/lower tension, and cool colors indicate lower ratios/higher tension.

3. Use one fixed color scale within each time series and for panels shown together or directly compared. A narrower scale may be used to display limited dynamic range only when this is stated explicitly; do not compare colors across panels that use different scales.

4. For a line-based heatmap, sample the ratio along the same oriented line at each time point, arrange position along the x-axis and time along the y-axis, and display the resulting matrix with the selected fixed color scale. Manually redraw the trajectory as needed to follow the same structure while retaining its orientation. Orient trajectories from left to right along lamellipodia and cell margins, from base to tip along filopodia, and from distal to proximal along cell protrusions. In the original analysis, Gaussian smoothing was applied to the resulting position-by-time matrices, but the Gaussian σ, line width, sampling interval, and numerical color ranges were not standardized among heatmaps. No separate interpolation step was specified.

**Interpretation:** Do not compare absolute ratios between α-actinin and α-catenin indicators, between different microscopes, or between specimens acquired with different settings. Use within-indicator, within-configuration comparisons.

**D. Replicates, data presentation, and statistics**

1. Repeat cultured-cell experiments independently at least twice and key validation experiments three or more times. An independent experiment is defined as a separate culture and transfection performed on a different day. In each experiment, acquire 10–15 fields and analyze approximately 15–30 cells in total per condition. Cells, fields, and ROIs within an experiment are subsamples rather than independent experimental replicates. For mouse experiments, report the number of animals and the number of cells and ROIs obtained from each animal separately.

2. The published cardiomyocyte time-lapse analysis included 10 z-disc ROIs from two independent experiments (Figure 4F of [4]). These ROIs were nested within the two experiments and should not be interpreted as 10 independent biological replicates; the result is presented as descriptive evidence. Direct organ imaging was qualitative and was not subjected to group-level statistical analysis.

3. Present time-course data as mean line plots with 95% confidence intervals and show individual cell or ROI measurements as swarm plots overlaid on violin plots. Report exact sample sizes and independent experiment numbers, and the mapping of cells or ROIs to experiments or animals in the corresponding figure legends. Supplementary Data 1 preserves the published cell-level dataset in its original form; experiment-day or animal mappings and a separate exclusion log were not added retrospectively.

4. In the original study, independent samples t-tests were used for comparisons between two independent groups, Wilcoxon signed-rank tests for paired comparisons, and one-way ANOVA followed by Scheffé’s post hoc test for comparisons involving more than two groups. These statements describe the analyses reported in the original study [4]. In this protocol, longitudinal cell time courses shown in Figure 1 are presented descriptively because repeated measurements and nested subsamples were not modeled explicitly. For new inferential analyses, use experiment-level summaries or an appropriate mixed-effects model that accounts for repeated measurements and nesting.

## Validation of protocol

This protocol was validated in Fujiwara et al. [4]. The α-actinin indicator showed concentration-dependent increases in TS fluorescence and the relaxation ratio following treatment with 5–20 μM blebbistatin, whereas mCherry remained stable. Control constructs showed reduced or no responses, and fixed-cell controls showed no appreciable photobleaching (Figures 1E–F and 2A, and Supplementary Figure 1C–D of [4]).

The α-catenin indicator also responded to blebbistatin, whereas controls lacking the tension sensor linker or β-catenin-binding region showed reduced responses (Figure 3A and Supplementary Figure 3 of [4]). Time-lapse and direct ex vivo imaging resolved distinct tension patterns in cultured cells, heart, and liver (Figures 3B–K, 4B, and 5B–D of [4]).

In isolated cardiomyocytes, blebbistatin increased TS fluorescence and the relaxation ratio while mCherry remained stable; the analysis included 10 z-disc ROIs from two independent experiments (Figure 4D–F of [4]). Single-molecule optical-tweezer measurements further demonstrated force-dependent fluorescence changes between 0 and 6 pN and reversible sensor behavior (Figure 1G–K and Supplementary Figure 1F of [4]).

## General notes and troubleshooting

**General notes**

1. These indicators report relative changes in molecular loading, not absolute force. Because the α-actinin and α-catenin indicators sample different molecular complexes, their green/red ratios should not be compared directly.

2. Select cells with moderate, nonsaturated expression and the expected localization. Excessive expression can alter molecular stoichiometry, and excess α-catenin indicator may modestly underestimate tension by perturbing vinculin-dependent reinforcement [4].

3. In the original validation study [4], indicator responses were more readily resolved with the tested sCMOS and EMCCD systems than with the tested PMT system. This was an empirical observation and does not establish a general detector-class requirement. Regardless of detector type, quantitative analysis requires clearly detectable signals in both channels and the absence of saturated pixels; for 16-bit images, ROIs containing pixels at the digital maximum of 65,535 are excluded.

4. Inspect both raw channels together with ratio images, as low signal and chromatic misregistration can produce ratio artifacts.

5. Protect blebbistatin solutions from light and minimize illumination during imaging. Para-aminoblebbistatin can be used for prolonged imaging [8].

6. Before applying the indicators to a new cell type, confirm the expected localization and blebbistatin response, preferably using a no-linker or binding-deficient control.

7. Cardiomyocyte isolation requires training in Langendorff perfusion and an approved animal protocol.

8. Because BDM is maintained during cardiomyocyte imaging, evaluate blebbistatin responses relative to the pretreatment image acquired at the same BDM concentration rather than as an uninhibited physiological baseline.

**Troubleshooting**

**Problem 1:** No or weak TS signal.

Possible causes: Low expression or low transfection efficiency; suboptimal cell confluence; insufficient detector sensitivity; photobleaching.

Solutions: Confirm mCherry expression and plasmid identity. Transfect actively proliferating cells at approximately 80% confluence using Lipofectamine 2000 as described in Section B. Increase detector sensitivity before increasing laser power.

**Problem 2:** mCherry changes during a short drug response.

Possible causes: Focus drift caused by thermal instability or medium exchange; dish or stage movement; photobleaching.

Solutions: Thermally equilibrate the imaging system and sample, secure the dish on the stage, and minimize disturbance during medium exchange. Use focus stabilization and minimize excitation intensity and exposure time.

**Problem 3:** No apparent blebbistatin response.

Possible causes: Inactive or light-damaged drug; incorrect concentration; cell-to-cell variability or poor cell condition; indicator mislocalization; limited detector sensitivity; cell type–dependent response kinetics.

Solutions: Prepare a fresh, light-protected working solution and first test 15 μM blebbistatin. Verify the dilution and indicator localization. Analyze multiple cells, as some may show little or no response. Quantify the TS/mCherry ratio rather than relying solely on visual inspection, especially for images acquired with photomultiplier-tube (PMT) detectors. Acquire images at 1-min intervals for at least 5 min and extend the observation period for slowly responding cell types.

**Problem 4:** Diffuse green background and fluorescent precipitates developing after blebbistatin addition.

Possible cause: Precipitation and intrinsic fluorescence of blebbistatin in aqueous medium.

Solution: Confirm that the blebbistatin stock has completely redissolved, prepare the working solution immediately before use in medium equilibrated to the imaging temperature, and minimize 488-nm illumination. Inspect cell-free regions to distinguish drug fluorescence from the sensor response. If precipitation or background fluorescence persists, use nonfluorescent para-aminoblebbistatin [8].

**Problem 5:** Red and green signals are spatially offset, producing ratio artifacts at structure edges.

Possible causes: Chromatic misregistration caused by wavelength-dependent chromatic aberration; sample movement between sequential channel acquisitions.

Solution: Correct the chromatic shift using Chromagnon [10]. If sample movement is suspected, minimize the interchannel delay and stabilize the specimen.

**Problem 6:** Highly noisy ratio map.

Possible causes: Weak mCherry signal; division by background-dominated pixels; detector noise.

Solutions: Increase the mCherry signal without saturation, use a sensitive detector with a wide dynamic range, and interpret only regions with reliable signals in both raw channels.

**Problem 7:** Poor cardiomyocyte morphology.

Possible causes: Over/under-digestion, harsh trituration, poor coating, or delayed processing.

Solutions: Optimize collagenase activity and endpoint, use a large-bore pipette, minimize handling, and prepare fresh laminin-coated dishes.

## Supplementary information

The following supporting information can be downloaded here:

1. Supplementary Data 1: Numerical source data

2. Supplementary Data 2: Plasmid DNA sequences

3. Supplementary Movie 1. Lamellipodia dynamics visualized with the α-actinin tension indicator.

4. Supplementary Movie 2. Filopodia dynamics visualized with the α-actinin tension indicator.

5. Supplementary Movie 3. Cell protrusion dynamics visualized with the α-catenin tension indicator.

6. Supplementary Movie 4. Cell margin dynamics visualized with the α-catenin tension indicator.

7. Supplementary Movie 5. Cardiomyocyte expressing the α-actinin tension indicator.

8. Supplementary Movie 6. Magnified view of the cardiomyocyte expressing the α-actinin tension indicator.
